# Supplementary material for: A machine learning algorithm to increase COVID-19 inpatient diagnostic capacity
Source: PLoS One. 2020 Sep 22;15(9):e0239474. doi: 10.1371/journal.pone.0239474 (PMC7508387; doi:10.1371/journal.pone.0239474)
Supplement: S1 Checklist — (PDF) [file pone.0239474.s001.pdf]

| Section & Topic | No | Item | Reported on page # |
|---|---|---|---|
| **TITLE OR ABSTRACT** | | | |
| | **1** | Identification as a study of diagnostic accuracy using at least one measure of accuracy (such as sensitivity, specificity, predictive values, or AUC) | Page 1: A machine learning algorithm to increase COVID-19 inpatient diagnostic capacity |
| **ABSTRACT** | | | |
| | **2** | Structured summary of study design, methods, results, and conclusions (for specific guidance, see STARD for Abstracts) | Page 2 |
| **INTRODUCTION** | | | |
| | **3** | Scientific and clinical background, including the intended use and clinical role of the index test | Page 3: Introduction |
| | **4** | Study objectives and hypotheses | Our aim was to develop a machine learning algorithm using the largest dataset to date, to serve as a COVID-19 diagnostic proxy to be useful in hospitals where SARS-CoV-2 specific PCR testing is unavailable or scarce. We hypothesized that a machine learning-based algorithm based on a parsimonious set of blood markers that include inflammatory markers could predict the presence or absence of COVID-19 with high sensitivity and potentially be used as a screening tool in clinical practice. |
| **METHODS** | | | |
| *Study design* | **5** | Whether data collection was planned before the index test and reference standard were performed (prospective study) or after (retrospective study) | Page 8: We retrospectively considered all cases that were tested for SARS-CoV-2 in the emergency room or inpatient setting within the UCLA Health System between 1 March 2020 and 24 May 2020. |
| *Participants* | **6** | Eligibility criteria | Page 8: After constructing our initial pool of cases, we included only cases with complete blood counts and at least one inflammatory marker (C-reactive protein, ferritin, or LDH) within 48 hours of the sample collection for SARS-CoV2 PCR testing. |
| | **7** | On what basis potentially eligible participants were identified (such as symptoms, results from previous tests, inclusion in registry) | Page 8: See items 5 and 6 |
| | **8** | Where and when potentially eligible participants were identified (setting, location and dates) | Page 8: We retrospectively considered all cases that were tested for SARS-CoV-2 in the emergency room or inpatient setting within the UCLA Health System between 1 March 2020 and 24 May 2020. |
| | **9** | Whether participants formed a consecutive, random or convenience series | Page 4: We retrospectively considered all cases that were tested for SARS-CoV-2 |
| *Test methods* | **10a** | Index test, in sufficient detail to allow replication | Page 9: Features in the models were age, gender, hemoglobin, red blood cell count, absolute neutrophil, absolute lymphocyte, absolute eosinophil and absolute basophil counts, the neutrophil to lymphocyte ratio, C-reactive protein, ferritin, and LDH. Page 10: An ensemble (combined) model was then created based on those seven individually trained machine learning models. The final classification as positive or |

CC BY SA

| | | | | |
|---|---|---|---|---|
| | | | negative was decided using the majority vote of the classifiers calculated by averaging their respective probabilities. | |
| | **10b** | Reference standard, in sufficient detail to allow replication | Page 9: Diagnosis of SARS-CoV-2 was confirmed by PCR testing assays performed at the UCLA Microbiology Laboratory. These assays included the 2019-nCoV Real-Time (RT)-PCR Diagnostic Panel (CDC, Atlanta, Georgia), the Diasorin Simplexa COVID-19 Direct RT-PCR (Diasorin Molecular LLC, Cypress, CA), the TaqPath COVID-19 Combo Kit (Thermo Fisher Scientific Inc., Waltham, MA). | |
| | **11** | Rationale for choosing the reference standard (if alternatives exist) | No suitable alternative exists | |
| | **12a** | Definition of and rationale for test positivity cut-offs or result categories of the index test, distinguishing pre-specified from exploratory | Page 10: The discriminatory operating threshold was determined using a validation set held out from the training set and selected such that the sensitivity on the validation set would be above a predefined threshold of 0.95 by configuring the beta parameter of the F-score. | |
| | **12b** | Definition of and rationale for test positivity cut-offs or result categories of the reference standard, distinguishing pre-specified from exploratory | Not applicable | |
| | **13a** | Whether clinical information and reference standard results were available to the performers/readers of the index test | Not applicable. Reference standard needed for model training. However, machine learning model predictions were performed on held out test set. | |
| | **13b** | Whether clinical information and index test results were available to the assessors of the reference standard | Not applicable. | |
| *Analysis* | **14** | Methods for estimating or comparing measures of diagnostic accuracy | Page 10: The resulting model was then evaluated on the held-out test set using the following diagnostic metrics: area under the receiver operator curve (AUROC), area under the precision recall curve (AUPRC), sensitivity, specificity, negative predictive value (NPV), and positive predictive value (PPV). Confidence intervals were constructed for each metric using a bootstrapping procedure on the test set in which the test set was repeatedly resampled with replacement 1000 times. | |
| | **15** | How indeterminate index test or reference standard results were handled | Not applicable. All tests were either positive or negative. | |
| | **16** | How missing data on the index test and reference standard were handled | Page 9: The normalization parameters (e.g., mean and standard deviation) were computed using the training set, and the features in the test set were scaled using these values. After scaling, missing lab values were imputed with zero, effectively inserting the mean feature value from the training set. Mean imputation was determined appropriate after evaluating several imputation methods (K-nearest neighbor and Iterative Imputation), which did not result in significant improvements. | |

| | | | | |
|---|---|---|---|---|
| | **17** | Any analyses of variability in diagnostic accuracy, distinguishing pre-specified from exploratory | Page 10: To test the contribution of each feature to model performance, the feature values were randomly shuffled, thereby disrupting their correlations with the outcome, and the decrease in model performance (f1-score) was recorded. Page 5: In sensitivity analyses, we calculated AUROC and AUPRC when adding the inflammatory features relative to the baseline model of only demographic characteristics and features of the complete blood cell count (see Figure 4). | |
| | **18** | Intended sample size and how it was determined | Page 8: We retrospectively considered all cases that were tested for SARS-CoV-2 in the emergency room or inpatient setting within the UCLA Health System between 1 March 2020 and 24 May 2020. | |
| **RESULTS** | | | | |
| *Participants* | **19** | Flow of participants, using a diagram | Page 14: Figure 1 | |
| | **20** | Baseline demographic and clinical characteristics of participants | Page 6: Mean age was 58.1 (SD 22.3), 53% were men, 49% white, 24% Latino, and 29% immunosuppressed. | |
| | **21a** | Distribution of severity of disease in those with the target condition | N/A | |
| | **21b** | Distribution of alternative diagnoses in those without the target condition | N/A | |
| | **22** | Time interval and any clinical interventions between index test and reference standard | Page 4: we included only cases that had CBC counts and at least one inflammatory marker (C-reactive protein, ferritin, or LDH) within 48 hours of the sample collection for SARS-CoV2 testing. | |
| *Test results* | **23** | Cross tabulation of the index test results (or their distribution) by the results of the reference standard | Page 14: Figure 1 | |
| | **24** | Estimates of diagnostic accuracy and their precision (such as 95% confidence intervals) | Page 4: The AUROC of the model in the held-out test set (n=392) was 0.91 (95% confidence interval [CI] 0.87-0.96) and the AUPRC was 0.76 (95% CI 0.65-0.85). The model achieved a sensitivity of 0.93 (95% CI 0.84-0.98), specificity of 0.64 (95% CI 0.59-0.69), NPV of 0.98 (95% CI 0.96-1.00), and PPV of 0.29 (95% CI 0.23-0.36). Receiver operator curves and precision-recall curves were presented in Figure 2. | |
| | **25** | Any adverse events from performing the index test or the reference standard | N/A | |
| **DISCUSSION** | | | | |
| | **26** | Study limitations, including sources of potential bias, statistical uncertainty, and generalisability | Page 7: Our findings should be considered in light of the following limitations | |
| | **27** | Implications for practice, including the intended use and clinical role of the index test | Page 8: This machine learning modality may be especially useful as a screening test in smaller medical centers or those in resource-poor regions that may have limited capacity for COVID-19 PCR-based diagnosis, or in instances were testing capacity is in danger due to low supplies. | |
| **OTHER INFORMATION** | | | | |
| | **28** | Registration number and name of registry | N/A | |

| | | | | |
|---|---|---|---|---|
| | **29** | Where the full study protocol can be accessed | Page 11, algorithm will be publicly available. | |
| | **30** | Sources of funding and other support; role of funders | Page 11 | |

# STARD 2015

## AIM

STARD stands for “Standards for Reporting Diagnostic accuracy studies”. This list of items was developed to contribute to the completeness and transparency of reporting of diagnostic accuracy studies. Authors can use the list to write informative study reports. Editors and peer-reviewers can use it to evaluate whether the information has been included in manuscripts submitted for publication.

## EXPLANATION

A **diagnostic accuracy study** evaluates the ability of one or more medical tests to correctly classify study participants as having a **target condition.** This can be a disease, a disease stage, response or benefit from therapy, or an event or condition in the future. A medical test can be an imaging procedure, a laboratory test, elements from history and physical examination, a combination of these, or any other method for collecting information about the current health status of a patient.

The test whose accuracy is evaluated is called **index test.** A study can evaluate the accuracy of one or more index tests. Evaluating the ability of a medical test to correctly classify patients is typically done by comparing the distribution of the index test results with those of the **reference standard**. The reference standard is the best available method for establishing the presence or absence of the target condition. An accuracy study can rely on one or more reference standards.

If test results are categorized as either positive or negative, the cross tabulation of the index test results against those of the reference standard can be used to estimate the **sensitivity** of the index test (the proportion of participants *with* the target condition who have a positive index test), and its **specificity** (the proportion *without* the target condition who have a negative index test). From this cross tabulation (sometimes referred to as the contingency or “2x2” table), several other accuracy statistics can be estimated, such as the positive and negative **predictive values** of the test. Confidence intervals around estimates of accuracy can then be calculated to quantify the statistical **precision** of the measurements.

If the index test results can take more than two values, categorization of test results as positive or negative requires a **test positivity cut-off**. When multiple such cut-offs can be defined, authors can report a receiver operating characteristic (ROC) curve which graphically represents the combination of sensitivity and specificity for each possible test positivity cut-off. The **area under the ROC curve** informs in a single numerical value about the overall diagnostic accuracy of the index test.

The **intended use** of a medical test can be diagnosis, screening, staging, monitoring, surveillance, prediction or prognosis. The **clinical role** of a test explains its position relative to existing tests in the clinical pathway. A replacement test, for example, replaces an existing test. A triage test is used before an existing test; an add-on test is used after an existing test.

Besides diagnostic accuracy, several other outcomes and statistics may be relevant in the evaluation of medical tests. Medical tests can also be used to classify patients for purposes other than diagnosis, such as staging or prognosis. The STARD list was not explicitly developed for these other outcomes, statistics, and study types, although most STARD items would still apply.

## DEVELOPMENT

This STARD list was released in 2015. The 30 items were identified by an international expert group of methodologists, researchers, and editors. The guiding principle in the development of STARD was to select items that, when reported, would help readers to judge the potential for bias in the study, to appraise the applicability of the study findings and the validity of conclusions and recommendations. The list represents an update of the first version, which was published in 2003.

More information can be found on http://www.equator-network.org/reporting-guidelines/stard.

cc BY SA
